# ReCOV: recovery and rehabilitation during and after COVID-19 – a study protocol of a longitudinal observational study on patients, next of kin and health care staff

**DOI:** 10.1186/s13102-021-00299-9

**Published:** 2021-06-30

**Authors:** E. Rydwik, L. Anmyr, M. Regardt, A. McAllister, R. Zarenoe, E. Åkerman, Y. Orrevall, M. Bragesjö, O. Dahl, M. K. Kemani, L. Nordstrand, U. Ekman, L. Holmström, M. Nygren-Bonnier

**Affiliations:** 1 Department of Neurobiology, Care Sciences and Society, Division of Physiotherapy, Karolinska Institutet, Huddinge, Sweden; 2 Women’s Health and Allied Health Professionals Theme, Medical Unit Occupational Therapy and Physiotherapy, Karolinska University Hospital, Solna, Sweden; 3 Women’s Health and Allied Health Professionals Theme, Department of Social Work in Health, Karolinska University Hospital, Solna, Sweden; 4 Department of CLINTEC, Karolinska Institutet, Stockholm, Sweden; 5 Department of Neurobiology, Care Sciences and Society, Division of Occupational Therapy, Karolinska Institutet, Huddinge, Sweden; 6 Women’s Health and Allied Health Professionals Theme, Medical Unit Speech and Language Pathology, Karolinska University Hospital, Stockholm, Sweden; 7 CLINTEC, Division of Speech-Language Pathology, Karolinska Institutet, Stockholm, Sweden; 8 Perioperative Medicine and Intensive Care Function, Department of Intensive Care, Karolinska University Hospital, Stockholm, Sweden; 9 Department of Neurobiology, Care Sciences and Society, Division of Nursing, Karolinska Institutet, Huddinge, Sweden; 10 Women’s Health and Allied Health Professionals Theme, Medical Unit Clinical Nutrition, Karolinska University Hospital, Stockholm, Sweden; 11 Department of Biosciences and Nutrition, Karolinska Institutet, Stockholm, Sweden; 12 Department of Clinical Neuroscience, Division of Psychology Karolinska Institutet, Stockholm, Sweden; 13 Women’s Health and Allied Health Professionals Theme, Medical Unit Medical Psychology Karolinska University Hospital, Solna, Sweden; 14 Department of Psychology, Stress Research Institute, Stockholm University, Stockholm, Sweden; 15 Department of Neurobiology, Care Sciences and Society, Division of Clinical Geriatrics, Karolinska Institutet, Huddinge, Sweden

**Keywords:** Infection, Physical function, Well-being

## Abstract

**Background:**

The knowledge of the long-term consequences of covid-19 is limited. In patients, symptoms such as fatigue, decreased physical, psychological, and cognitive function, and nutritional problems have been reported. How the disease has affected next of kin, as well as staff involved in the care of patients with covid-19, is also largely unknown. The overall aim of this study is therefore three-fold: (1) to describe and evaluate predictors of patient recovery, the type of rehabilitation received and patients’ experiences of specialized rehabilitation following COVID-19 infection; (2) to study how next of kin experienced the hospital care of their relative and their experiences of the psychosocial support they received as well as their psychological wellbeing; (3) to describe experiences of caring for patients with COVID-19 and evaluate psychological wellbeing, coping mechanisms and predictors for development of psychological distress over time in health care staff.

**Methods:**

This observational longitudinal study consists of three cohorts; patients, next of kin, and health care staff. The assessments for the patients consist of physical tests (lung function, muscle strength, physical capacity) and questionnaires (communication and swallowing, nutritional status, hearing, activities of daily living, physical activity, fatigue, cognition) longitudinally at 3, 6 and 12 months. Patient records auditing (care, rehabilitation) will be done retrospectively at 12 months. Patients (3, 6 and 12 months), next of kin (6 months) and health care staff (baseline, 3, 6, 9 and 12 months) will receive questionnaires regarding, health-related quality of life, depression, anxiety, sleeping disorders, and post-traumatic stress. Staff will also answer questionnaires about burnout and coping strategies. Interviews will be conducted in all three cohorts.

**Discussion:**

This study will be able to answer different research questions from a quantitative and qualitative perspective, by describing and evaluating long-term consequences and their associations with recovery, as well as exploring patients’, next of kins’ and staffs’ views and experiences of the disease and its consequences. This will form a base for a deeper and better understanding of the consequences of the disease from different perspectives as well as helping the society to better prepare for a future pandemic.

## Background

COVID-19 was declared a global pandemic in March 2020 by the World Health Organisation (WHO) [1]. So far (May, 2021), there are over 1 000,000 confirmed cases and more than 14,000 deaths in Sweden [2]. Initially, it was assumed that COVID-19 would primarily affect the airways, but several studies have now shown that it is an infection with multisystem manifestations. The impact of the virus ranges from an asymptomatic infection to a very severe illness and signs and symptoms may arise from the cardiac, renal gastrointestinal, nervous, endocrine, and musculoskeletal systems and may change over time [3, 4]. Therefore, COVID-19 has the potential to affect physical, cognitive, and psychological functions in multiple ways. It has been clear that a significant proportion of patients with COVID-19 develop long-term symptoms [3–10].

Previous research has shown that a prolonged stay at an intensive care unit (ICU) is associated with a significant negative impact on lung function, voice function, nutritional status and physical function, intensive care delirium and decreased psychological health. These symptoms can persist and have a negative impact on quality of life in the long-term perspective [5, 6, 11]. However, post-acute COVID-19 symptoms may also occur after so-called mild COVID-19 [5]. An increasing number of patients that never required hospitalization during their COVID-19 illness are displaying the same long-term symptoms, of varying severity, involving several organ systems similar to hospital treated patients [6, 7, 11]. These long-term symptoms in non-hospitalized patients have significant impact on their performance of daily activities as well as the ability to return to work [7, 11]. In addition, impaired physical and psychological function as well as varying degrees of fatigue and cognitive impairment can led to a decreased health-related quality of life [4–7].

Due to the isolation of patients and not allowing visits from relatives, specific telephone lines for relatives were created at Karolinska University Hospital in Stockholm to relieve the staff at the wards; something that has never been done in Sweden before. The support line was staffed by medical social workers, two psychologists and a nurse and were open during days and evenings, 7 days a week. The medical social workers proactively contacted relatives after the patients had been admitted to the hospital, to offer support to their relatives regarding social- and psychosocial situation, and a social anamnesis was obtained. The medical social workers also contacted relatives in the event of death. At first, relatives were given the opportunity to say a final farewell via digital media when the patients were in the end-of-life stage, but at the end of wave 1, relatives could visit for a final farewell. Few studies exist regarding this type of support for relatives in similar situations. During the SARS epidemic in Hong Kong, a “hotline” and a help desk were staffed where relatives could turn for support and information [12]. The intervention was only evaluated through a short questionnaire that was handed out to close relatives who used the helpdesk function for a week. The results were positive, but what the residual effect may be remains unanswered. One function that was requested for the future was the possibility of communication via digital media.

Karolinska University Hospital has cared for the largest number of patients with severe COVID-19 in Sweden during 2020. The hospital’s employees have worked under extraordinary conditions, which has raised concerns about lack of protective equipment, long work shifts, high workload, moral stress, fear of infection, exposure to potentially traumatic experiences and lack of opportunities for recovery. In a study published early in the ongoing pandemic, the results showed that health care professionals in close contact with patients with COVID-19 in Wuhan, and other severely affected regions of China, reported major psychological strains, including depression, anxiety, difficulty sleeping and post-traumatic stress symptoms [13, 14]. Similar reports, on short term psychological distress in health care staff have also been published from Italy and the US during the spring [15–17]. Previous experiences from the SARS epidemic in 2003 show that in addition to developing disorders in the short term, healthcare professionals also risk long-term psychological distress. Long-term follow-up, up to 2 years after the SARS epidemic showed that healthcare professionals caring for critically ill patients had an increased risk of burnout, anxiety, post-traumatic stress disorder and short-term sick leave compared to healthcare professionals from a nearby city not affected by the virus to the same extent [18, 19].

In summary, the knowledge of consequences of being severely ill and treated in hospital, with or without intensive care due to COVID-19 is limited. Consequences such as fatigue, decreased physical and psychological function have been reported, but little is known of the long-term consequences. How the pandemic has affected next of kin and staff are also at large unknown.

**Fig. 1:**
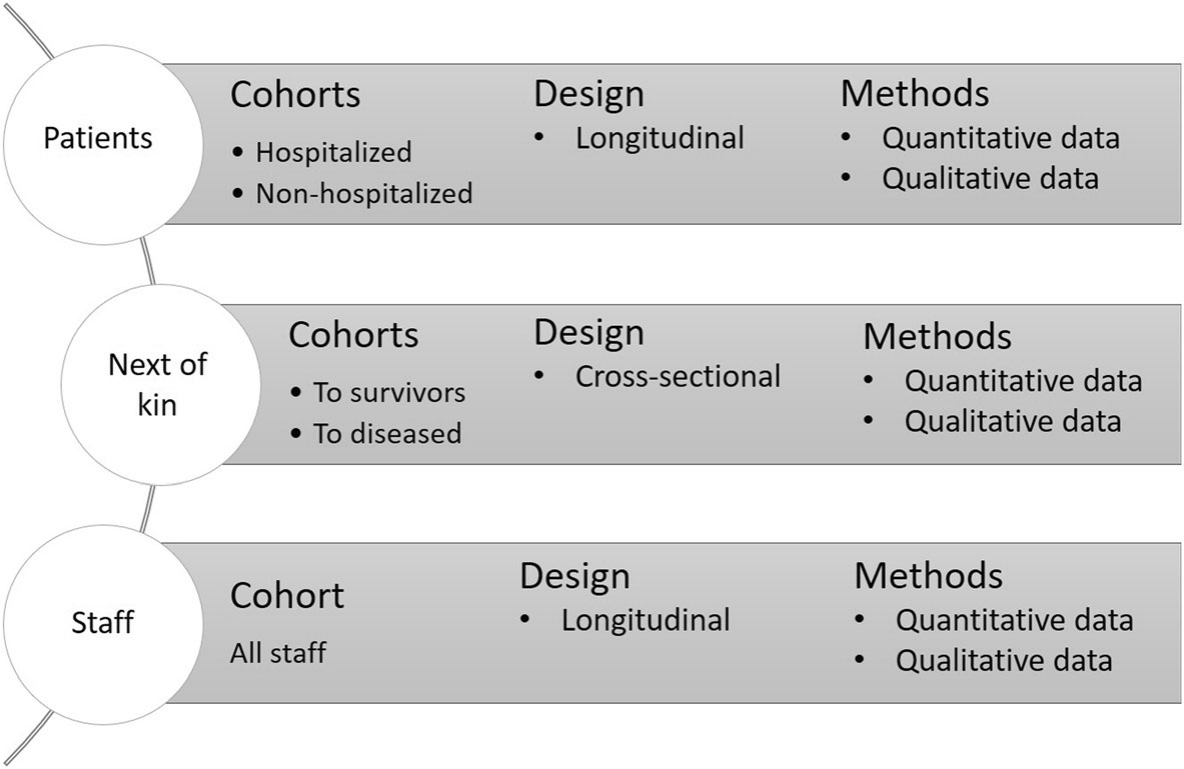
Description of the cohorts

This observational and longitudinal study consists of three cohorts; patients, next of kin (both those where their relative survived and those that lost their relative), and health care staff (Fig. 1).

Based on these three cohorts, the overall aims of this project are:

- to evaluate recovery and predictors of recovery in multiple health related areas such as physical function, cognition, fatigue, health-related quality of life and psychological health at 3, 6 and 12 months for patients with COVID − 19. The aim is also to describe type of care and rehabilitation as well as to explore how patients experienced their disease period and the care and rehabilitation they received.
- to study the psychological health of next of kin and how they experienced the hospital care and their experiences of the psychosocial support they received as well as communication with their relative and the staff.
- to describe experiences of caring for patients with COVID-19 and evaluate psychological wellbeing, coping mechanisms and predictors for development of psychological distress over time in health care staff.

## Methods

This study is a collaboration between the departments of Allied Health Professionals, Infection, Respiratory Medicine, Cardiology, Radiology and Intensive Care at the Karolinska University Hospital in Stockholm, Sweden.

The study will collect both quantitative and qualitative data. Quantitative data will be collected in an observational study with an explorative hypothesis-generating design in three cohorts. To capture experiences from patients, next of kin and health care staff, a qualitative design will be used. The designs in the different cohorts are described below and an overview is shown in Fig. 1.

### The patient cohort

**Fig. 2:**
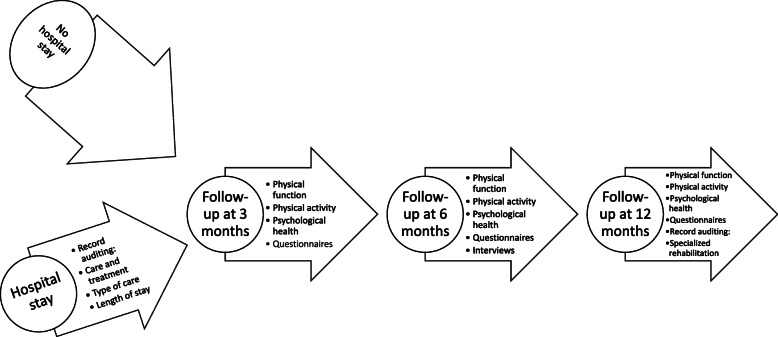
Process for collecting data in the longitudinal design for the patient cohort

This cohort include two groups, those that have been hospitalized and those who have been referred from primary care to the out-patient clinic due to long-term symptoms but were never hospitalized during the infection. The test battery consists of physical tests and screening, questionnaires, and interviews as well as patient records auditing (Table 1). The hospitalized patients will be followed longitudinally at 3, 6 and 12 months after discharge at an out-patient clinic at the hospital. At 12 months a retrospective auditing of patients’ record will be done to evaluate type and the effects of specialized rehabilitation during and after discharge (Fig. 2).

**Table 1:** Overview of measurements used in the different cohorts

Variables	Patients	Next of kin	Staff
Six-minute walk	x		
Chair-stand	x		
Hand grip strength	x		
Spirometry	x		
Inspiratory and expiratory muscle strength	x		
Activities of daily living	x		
Cognition	x		
Speech and voice	x		
Swallowing and eating	x		
Risk of malnutrition	x		
Hearing and Tinnitus	x		
Health-related Quality of Life	x	x	x
Health literacy	x		
Work ability	x	x	x
Fatigue	x	x	x
Sleeping disorders	x	x	x
Anxiety and depression	x	x	x
Post-traumatic stress	x	x	x
Burn-out			x

Patients above the age of 18 that are referred to the out-patient clinic will be asked to participate in the research project. This includes the following groups: 1) patients that have been hospitalized, age > 18, treated at ICU and/or at a ward requiring, high-flow oxygen, radiographic changes and/or a complicated clinical process; and 2) those who are referred from primary care with COVID-19-related symptoms from several organ system that have lasted for at least 3 months and that they are still on sick leave. Those that were discharged to nursing homes are excluded. The out-patient clinic will be coordinated together with the other departments at the hospital to reduce the burden on the patients.

#### Assessments

All patients that consent to participate will be screened and evaluated for physical and cognitive function, activities of daily living and work ability (those who worked before they got the disease), risk of malnutrition, hearing, voice- and swallowing disorders as well as psychosocial support. The following measurements and questionnaires will be used:

- Physical capacity: Six-minute walk (meters completed during 6 min) [20]
- Lower extremity strength: Chair stand 30-s and 1 min sit to stand [21]
- Hand grip strength: Jamar Hand dynamometer [22]
- Lung function: Micro-Loop [23]
- Inspiratory and expiratory muscle strength: Micro-RPM [24]
- Physical Activity: Frändin/Grimby scale [25] and Accelerometer (ActivePAL) [26]
- Activities of daily living: Canadian Occupational Performance Measure [27]
- Cognitive Function: Montreal Cognitive Assessment (MoCA) [28]
- Speech and voice: Self-assessment of acquired speech disorders (In Swedish: SOFT - Självsvarsformulär om förvärvade talsvårigheter) [29]
- Swallowing, eating and oral motor function: EAT10 [30], Nordic Orofacial Test-Screening [31]
- Screening for risk of malnutrition: Tool developed by the Swedish National Board of Health and Welfare [32]
- Hearing: The Hearing Handicap Inventory for the Elderly [33] and Tinnitus Handicap Inventory [34]
- Health-related Quality of Life: RAND-36 [35] and EQ-5D [36]
- Health literacy: eHEALS [37]
- Work ability: Work Ability Index [38]
- Fatigue: Mental Fatigue Scale [39], Fatigue Severity Scale [40]
- Sleeping disorders: Insomnia Severity Index [41]
- Anxiety and depression: Generalised Anxiety Disorder 7-item scale (GAD-7) [42], The Short Health Anxiety Inventory [43] and Patient Health Questionnaire-9 (PHQ-9) [44]
- Post-traumatic stress symptoms: Posttraumatic Stress Disorder Checklist for DSM-5 (PCL-5) [45], for patients cared for at ICU: Impact of Events Scale-6 (IES-6) [46]

#### Interviews

We will explore patients views of treatment, care and rehabilitation as well as their perceptions of psychosocial support, communication between health care professional, patients and next of kin as well as emotional consequences of the disease period. A strategic and purposive sample in consideration to sex, age, sociodemographic factors and disease severity will be invited to participate in semi-structured interviews. The interviews will take place at different time points after onset of disease as well as in relation to the first, second and third wave. A fourth group will consist of non-hospitalized patients. Each group will include approximately 20 participants. The participants will decide if they want a relative present or not.

They will be asked for participation through a letter by mail or with a telephone call. The participants can choose whether they want to be interviewed by phone, digital meeting or a physical meeting at the hospital and the interviews are estimated to take 30–60 min. The interview guide will be piloted during the 2–3 first interviews and modified if necessary. Transcripts and participation checking will be done if clarifications will be deemed necessary or if the participants request this. Data saturation will be considered during the recruitment period.

The interviewers (men and women) will have different professions, but they are all part of the rehabilitation team and they have previous experience of interviewing in qualitative research studies. The interviewers are employed by the hospital, but they do not have a caregiver- patient relationship. Before consenting to participate, the patients will be informed about the aim of the study and reasons for the interviewer to conduct them, i.e. increase the knowledge about this new disease and its consequences.

### The next of kin cohort

This cross-sectional cohort consists of two groups, one group that had a relative that survived and one with a relative that died. They will be asked to fill out questionnaires about background data such as age, sex as well as health-related quality of life, anxiety, depression, post-traumatic stress, work ability and sleeping disorders (described above) (Table 1). A strategic and purposive sample will be invited to participate in semi-structured interviews to explore their views and perceptions of psychosocial support and communication between health care professional, patients and next of kin.

For the next of kin with relative that died, an information letter and a request for participation will be sent together with the questionnaires. If they return the questionnaires, they consent to participate. A strategic sample of will then be contacted by a medical social worker to ask for consent to participate in an interview and approximately 20 people will be included.

When a patient that has been hospitalized is enrolled in the patient cohort, he/she is asked if we can contact their relatives, approximately 20 people will be included, consecutively. The procedure for enrolling relatives will follow the same procedure as described above.

The participants can choose whether they want to be interviewed by phone, a digital meeting or a physical meeting at the hospital and the interviews are estimated to take 30–60 min. The interview guide will be piloted during the 2–3 first interviews and modified if necessary. Transcripts will be returned to the participants if clarifications will be deemed necessary or if the participants request this. Data saturation will be considered during the recruitment period.

The interviewers (women) will be medical social workers or nurses and they have previous experience of interviewing in qualitative research studies. The interviewers are employed by the hospital, but they have not been involved in care or treatment for the next of kin or their relative. Before consenting to participate, the next of kin will be informed about the aim of the study and reasons for the interviewer to conduct them, i.e. increase the knowledge about how this new disease has affected next of kin.

### The staff cohort

A purposive sample of health care staff including nurses, assistant nurses, doctors, and allied health care professionals) working in intensive care units and wards where patients with COVID-19 have been cared for, or in other ways involved in the care of COVID-19 patients, at Karolinska University Hospital will be invited to participate via several internal communication channels (like the intranet, e-mail, and information letters). The study is longitudinal with five data collection points over 1 year.

#### Assessments

**Fig. 3:**
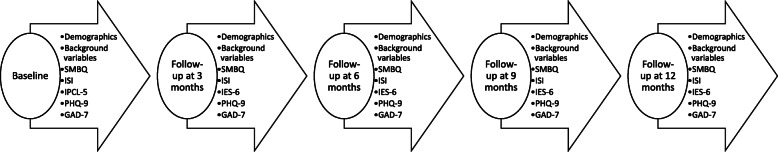
Process for collecting data in the longitudinal design for the health care staff cohort. Abbreviations: *SMBQ* Shirom-Melamed burnout questionnaire, *ISI* insomnia severity index, *PHQ-9* PCL-5 patient health questionnaire-9, GAD-7 generalized anxiety disorder

Assessments are conducted in intervals of 3 months, starting during the third quarter of 2020, i.e. baseline, and 3, 6, 9 and 12 months following baseline (see Fig. 3). Demographics and background variables (e.g., age, gender, marital status, professional affiliation, specialty, years in the profession, previous periods of mental illness and working conditions during the study period), and information from reliable and validated psychological health self-assessment forms are collected via an electronic platform. In addition to the self-report questionnaires distributed to patient and next of kin we also use the (see also Table 1):

- Burnout: Shirom-Melamed Burnout Questionnaire (SMBQ) [47]
- Coping Strategies: Coping Strategies Questionnaire (CSQ) [48]

#### Interviews

A strategic and purposive sample will be invited to participate in semi-structured interviews (individual interviews or focus groups) to explore their views and perceptions of caring for and treating patients with COVID-19. The interview guide will contain question areas about how one experienced the work during the pandemic, important experiences and what has been helpful in the form of staff support efforts, experiences of team work; experiences of existing and needed support and information; and experiences of how communication with colleagues, managers and patients has worked and what support one feels one needs going forward, as well as questions that capture psychological, emotional and practical experiences in relation to caring for patients with COVID-19.

The participants can choose whether they want to be interviewed by phone, digital meeting or a physical meeting at the hospital, and if they want to participate in individual interviews or focus groups. The interviews are estimated to take 30–60 min and the focus groups 60 min. The interview guides will be piloted during the 2–3 first interviews and modified if necessary. Field notes will be taken during the focus groups. Transcripts will be returned to the participants if clarifications will be deemed necessary or if the participants request this.

The interviewers (men and women) will be psychologists and medical social workers and they have previous experience of interviewing in qualitative research studies. The interviewers are employed by the hospital, but they have not been working together with the respondents. Before consenting to participate, respondents will be informed about the aim of the study and reasons for the interviewer to conduct them, i.e. increase the knowledge about how the pandemic has affected the staff.

When considering the sample size, we have taken into account the following aspects: (a) the aim of the study, (b) sample specificity, (c) use of established theory, (d) quality of dialogue, and (e) analysis strategy as proposed by Malterud et al. 2016 [49] and focused on the information power rather than saturation per se. The following factors, specific to this study, have been taken into consideration:

1. The fairly wide scope of the study aim and the topics covered by the semi structured interview guide
2. The sample including staff from different professions, disciplines (doctors, nurses, physiotherapists, psychologists, counselors, health care practitioners, occupational therapists, speech and language pathologists, dietitians etc.)
3. The wide range in demographics in the sample
4. The unique and extreme working environment imposed by the pandemic and the urgent need to capture a large variety in experiences.

These considerations combined has led us to an approximated sample of 80–100 participants.

### Analyses

Quantitative data will be described with mean (standard deviation), median (inter-quartile range) and proportion as well as parametric and non-parametric analyses depending on data level. Different regression analyses will be used depending on the research question and data level of the outcome measures. The longitudinal data will for example be analysed using Linear Mixed Models that in addition to studying change at the group level also can model change on the individual level, handle dependency for repeated observations and provide correct estimates with missing data.

The interviews will be transcribed in their entirety (verbatim) and analysed through an inductive content analysis that includes the following steps; repeated reading, coding of meaningful passages, categorization and sub-categorization [49, 50]. Experienced researchers in the project group will be responsible for the coding process and synthesizing the results in the different cohorts (mainly MN-B, LA, LH, MK, EÅ, OD). Mixed method analyses will also be used when combining quantitative and qualitative data.

## Preliminary results

The recruitment process is ongoing, below we describe the process and the recruitment rate so far.

### Patient cohort

So far approximately 1000 patients have been followed-up at the out-patient clinic, 750 that were hospitalized and 250 that are referred from primary care. Of those approximately 480 patients have given written informed consent to participate in the study. The 3-months follow-up has recently been finalized with patients from the first wave (March to June 2020). Six-months follow-up started in January 2021. The second wave (October to January/February 2021) of patients will have their first follow-up starting early spring of 2021. Interviews will start during spring 2021. Patients in the third wave (starting March 2021) will have their first follow-up starting June 2021.

### Next of kin cohort

Due to the second wave of the pandemic, recruitment had to be postponed but will start during late spring 2021.

### Staff cohort

Data collection is ongoing and began in August 2020, 430 staff members from several different professional categories (including nurses, assistant nurses, doctors, and allied health professions) have been included and are followed longitudinally. Analysis of the material collected from baseline will be carried out during the spring of 2021 and analysis of all data points will be carried out in the autumn of 2021. Recruitment to interviews is planned to be carried out during late spring 2021, or as soon as the third wave of the pandemic has mitigated and when healthcare staff are able to participate in interviews. Transcription and analysis of collected material will be carried out in 2022. Work on manuscripts will follow subsequently during 2023.

## Discussion

The rationale of this protocol article is to introduce a three-armed cohort study with the overarching aim to evaluate the consequences of COVID-19 in the perspective of the patients, the next of kin, and the health care staff.

COVID-19 is a new disease so there are large knowledge gaps that need to be filled. The project is highly relevant since the patient may have a slow recovery due to the severity of the disease. Empirical data indicate that the patients are affected on several levels and a rehabilitation period will be necessary to be able to return to their normal life, go back to work and to be able to participate in leisure activities and in the society again. This project will deepen the knowledge about the patient’s’ recovery, type of specialized rehabilitation as well as the patients and their next of kins’ experiences and views of the care- and rehabilitation process. It will also give knew knowledge about how the pandemic has affected psychological health among staff working with patients with COVID-19. The multidisciplinary nature of this project will help to cover the different types of problems that patients might experience and when necessary, patients will be offered specialized rehabilitation at the hospital’s out-patient clinic or referred to primary care when appropriate.

Several different tests and questionnaires are used in this study. As this is a new disease their psychometric properties have not been tested on this specific patient group. This can be regarded as a limitation. However, they are all tested for different psychometric properties in different patient groups [19–48], which can be regarded as sufficient. The fact that we used somewhat overlapping questionnaires in the three cohorts can be considered a strength since this will give possibilities to compare and evaluate psychological health and work ability across cohorts. The longitudinal design in combination with data retrieved from patient records will give us possibilities to describe, follow and describe the interventions the patients have been given in the clinical practice. Since this is a new disease and the currently available evidence is not sufficient to decide which intervention would be most appropriate to evaluate in a randomized controlled trial, an observational perspective is needed. In addition, the patients are inflicted with different long-term consequences and a multifactorial intervention is therefore most likely necessary. This can also be described and evaluated with an observational design.

In conclusion, this study will be able to answer different research questions both from a quantitative and qualitative perspective, by describing and evaluating long-term consequences and their associations with recovery as well as exploring patients, next of kin and staffs’ views and experiences of the pandemic. It will also generate knowledge to design and evaluate different interventions in future randomized controlled trials. This will form a base for a deeper and better understanding of the consequences of the disease from different perspectives (patients, next of kin and health care staff) which in turn will help the society to better prepare for a future pandemic.

## Data Availability

Data is not publicly available due to Swedish and European legislations, but available upon request. Requests for access to the data can be put to our Research Data Office (rdo@ki.se) at Karolinska Institutet and will be handled according to the relevant legislation. This will require a data processing agreement or similar with the recipient of the data.

## Abbreviations

ICU: Intensive care unit
MoCA: Montreal Cognitive Assessment
SOFT: Self-assessment of acquired speech disorders (In Swedish: Självsvarsformulär om förvärvade talsvårigheter)
EAT10: Eating assessment tool (10 items)
RAND-36: Measure of health-related quality of life
EQ-5D: EuroQol - 5 Dimension
eHEALS: eHEAlth Literacy Scale
GAD-7: Generalised Anxiety Disorder 7-item scale
PHQ-9: Patient Health Questionnaire-9
ISI: Insomnia Severity Index
PCL-5: Posttraumatic Stress Disorder Checklist for DSM-5
IES-6: Impact of Events Scale-6
SMBQ: Shirom-Melamed Burnout Questionnaire
CSQ: Coping Strategies Questionnaire
